# An improved PCR strategy for fast screening of specific and random integrations in rAAV-mediated gene targeted cell clones

**DOI:** 10.1186/1756-0500-4-246

**Published:** 2011-07-21

**Authors:** Yonglun Luo, Lars Bolund, Charlotte B Sørensen

**Affiliations:** 1 Department of Human Genetics, Institute of Biomedicine, Aarhus University, Aarhus, 8000, Denmark

## Abstract

**Background:**

Gene targeting by homologous recombination using recombinant adeno-associated virus (rAAV) is becoming a useful tool for basic research and therapeutic applications due to the remarkably high targeting frequency of rAAV virus vectors. However, the screening for the pure gene-targeted and random-integration-free primary cell clones is difficult since the cells have a limited proliferation capacity and often cannot be grown to produce sufficient DNA for non-PCR based analysis. This hampers the applications of this technology.

**Findings:**

In this study, we have developed an improved PCR screening method, which can be used for fast screening of clones with unwanted random integration (RI) of the rAAV genome. This improved screening method includes four PCRs: a PCR for the selection gene (e.g. Neo-PCR), a PCR for targeted gene knockout (e.g. BRCA1-KO-PCR), and two generalized PCRs for random integration of the rAAV genome (5'-AAV-RI-PCR, and 3'-AAV-RI-PCR). We have shown that this screening method greatly facilitates the procedure of screening for BRCA1 (BReast CAncer susceptibility gene 1) targeted cell clones, eliminating cell clones with both BRCA1 knockout and random integration of the rAAV genome.

**Conclusions:**

This screening method has facilitated the screening of correct gene-targeted cells. As the AAV-RI-PCRs are generalized PCRs, this method can also be applied for screening of rAAV-mediated targeting of other genes.

## Findings

**Figure 1:**
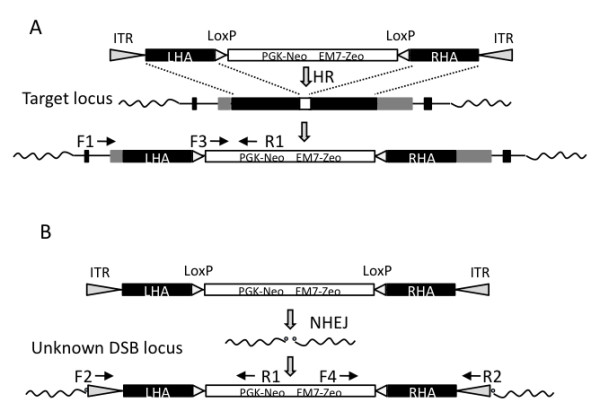
**Illustration of the targeting construct and screening PCRs**. Illustration of rAAV-mediated gene targeting by homologous recombination (A), and random gene integration of the rAAV genome by non-homologous end joining (B). Primers for screening PCRs of gene targeting and random integrations of the rAAV genome are indicated by arrows. ITR: inverted terminal repeat sequences; HR: homologous recombination; LHA: left homology arm; RHA: right homology arm; DSB locus: double-strand break locus; NHEJ: non-homologous end joining; EM7: bacterial EM7 promoter.

We have been using recombinant adeno-associated virus (rAAV)-mediated gene targeting to generate cloned pigs with specific gene knockout (KO) as models of human diseases [1]. However, screening for the pure gene knockout cell clones among the selection-positive cell clones can be difficult and hampers the applications of this technology. Primary fibroblasts are commonly used as target cells for gene targeting prior to cloning by somatic cell nuclear transfer. Unlike stem cells, these cells have a limited proliferation capacity rendering fast screening for knockout clones necessary. The standard screening strategy is based on PCR on cell lysates from cell clones (Figure 1A), and Southern blot analysis that requires long-term cultivation to obtain sufficient amounts of genomic DNA [1-3]. We have experienced that less than 50% of the selection-positive cell clones can be expanded sufficient for Southern blot screening when cultured in a normal growth medium (DMEM, +15%FCS, +P/S, +glutamine).

**Figure 2:**
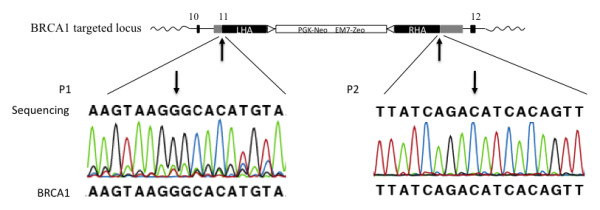
**Correct gene targeting verified by sequencing**. The correct 5' and 3' targeted boundary of one rAAV-mediated BRCA1 KO cell clone (5D1) was validated by sequencing. This BRCA1 KO cell clone has been used as nuclear donor cells for somatic cell nuclear transfer to generate BRCA1 KO pigs (Luo et al. Transgenic research 2010). Sequencing was performed with the primers P1 and P2.

In this study, we have developed an improved PCR screening method, which can be used for screening of clones with unwanted random integration (RI) of the rAAV genome (Figure 1B). During gene targeting by perfect homologous recombination, strand exchange occurs between the homology arms and the targeted locus (Figure 1A), without incorporation of the inverted terminal repeat sequences (ITR) of the virus. Sequencing of the 5' and 3' targeting sites of one of our BRCA1 KO clones confirms this (Figure 2). However, random integrations of the rAAV genome by non-homologous end joining will most often insert the viral genome (or parts hereof), including the ITR sequences, into the host cell genome. Based on the absence or presence of ITR sequences in cells with gene targeted or random integrations, respectively, we have designed a generalized rAAV random integration specific PCR assay [5'-AAV-RI-PCR (F2+R1) and 3'-AAV-RI-PCR (F4+R2)] by using a primer in the selection cassette (R1 or F4) and an ITR specific primer (F2 or R2), respectively (Figure 1B). We have to optimize the annealing temperature to 68°C to obtain satisfactory function of the ITR specific primer - probably due to the hairpin structure of the ITR element.

**Figure 3:**
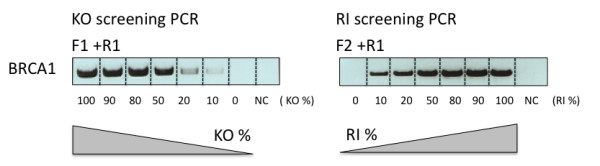
**The screening PCRs can semi-quantitatively detect the proportions of KO and RI DNA**. Semi-quantitative screening PCRs (BRCA1-KO-PCR (1952 bp) and 5'-AAV-RI-PCR (1943 bp)) on genomic DNA (50 ng) mixed from genomic DNA from BRCA1 knockout (KO) and random integration (RI) clones in a ratio of 100%, 90%, 80%, 50%, 20%, 10%, and 0% (KO: RI), respectively. NC, negative control. KO %, percentage of gene KO; RI %, percentage of random integration.

**Figure 4:**
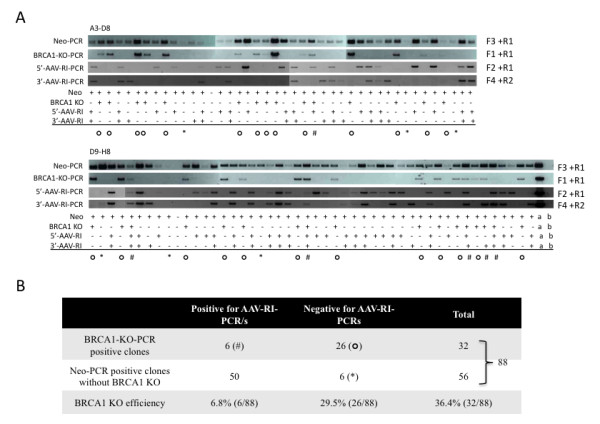
**Screening PCRs for BRCA1 KO in rAAV-mediated BRCA1 KO cells**. Screening PCRs (Neo-PCR (490 bp), BRCA1-KO-PCR (1952 bp), 5'-AAV-RI-PCR (1943 bp) and 3'-AAV-RI-PCR (1728 bp)) were performed using one μl cell lysate (A). Genotype summary of clones that are BRCA1 KO only (○), BRCA1 KO with random integration (#), random integration without the ITR sequences (*), and random integration with the ITR sequences (the rest) are listed in (B). "a", positive control (DNA, which is positive for the four screening PCRs); "b" negative control.

Using the KO specific PCR and the 5'-AAV-RI-PCR, we first tested the screening strategy on cells subjected to rAAV-mediated BRCA1 (BReast CAncer susceptibility gene 1) - KO [1]. Our results showed that these PCRs could semi-quantitatively distinguish the percentages of KO and RI in pooled DNA samples (50 ng genomic DNA) (Figure 3). We then applied this screening strategy to screen for rAAV-mediated BRCA1 KO cell clones from a 96-well plate. Ninety G418 resistant cell clones were harvested as previously described (Additional file 1). Cell lysates were used for Neo-PCR, BRCA1-KO-PCR, 5'-AAV-RI-PCR, and 3'-AAV-RI-PCR (Figure 4A). The screening results (Figure 4B) showed that eighty-eight clones (97.8%, 88/90) were positive for the Neo-PCR. Thirty-two out of the 88 Neo-PCR positive clones were positive for BRCA1-KO-PCR (KO efficiency = 36.4% (32/88)). Six of the 32 BRCA1 KO clones (6.8%) harbored random integrations of one or both of ITR sequences, and the remaining 26 clones (29.5%) were only positive for the BRCA1-KO-PCR. However, we also observed that six of the 56 clones, which were negative for BRCA1-KO-PCR, were negative for 5'- or 3'-AAV-RI-PCR as well, indicating that these six clones (10.7%, 6/56) might carry random integrations of a fragment without the ITR sequences. However, four of these six clones show a very weak Neo-PCR band, which indicates that these four samples have very low amounts of template. The negative BRCA1-KO-PCR and AAV-RI-PCR of these four clones could also result from too low amounts of temperate. Thus, among the 26 BRCA1-KO-PCR positive and AAV-RI-PCRs negative clones, we cannot be absolutely sure that there is no random insertion of the rAAV genome. Further screening by e.g. Southern blotting is required to genotype these clones. We have used this screening strategy to evaluate the results of rAAV-KO of other genes (ApoE in Yucatan fibroblasts and BRCA1 in Göttingen fibroblasts). The method has greatly facilitated the screening of gene-targeted cells and eliminates most of the KO clones, which carry random integrations, or heterogeneous clones of KO and RI cells. Detailed methods and primers used in this study are provided in Additional file 1 and Additional file 2 Table S1.

## Competing interests

The authors declare that they have no competing interests.

## Authors' contributions

YL participated in the design of the study, performed the experimental procedures and the data analysis, and wrote the manuscript. LB and CBS elaborated the study design, management, coordination, and drafting the manuscript. The authors have all read and approved the final manuscript.

## Supplementary Material

Additional file 1 **PCR screening methods**. details of PCR screening methods. Click here for file

Additional file 2 **Primers**. list of primers used in this study. Click here for file
