# Supplementary material for: An improved PCR strategy for fast screening of specific and random integrations in rAAV-mediated gene targeted cell clones
Source: BMC Res Notes. 2011 Jul 21;4:246. doi: 10.1186/1756-0500-4-246 (PMC3154164; doi:10.1186/1756-0500-4-246)
Supplement: Additional file 1 — PCR screening methods. details of PCR screening methods. [file 1756-0500-4-246-S1.DOC]

**Additional file 1**

*rAAV/BRCA1 KO virus transduction and G418 selection for G418-resistant cell clones*

Primary porcine fibroblasts (1 X 10^6^) were seeded into a gelatin-coated 10 cm cell culture dish 24 hours before transduction. The cells were kept in 3 ml fresh complete fibroblast growth medium (DMEM, +15%FCS, +P/S, +glutamine) and incubated with the virus particles, rAAV1/BRCA1 KO viral particles (5 X 10^5^ particles/cell), for 3 hours. Another seven ml complete fibroblasts growth medium was subsequently added to the cells. Twenty-four hours post transduction, the cells were trypsinized and seeded into 96-well plates at a dilution of one P10 dish into fifty 96-well plates, which would approximately give 400 cells per well. Three days post transduction the growth medium was supplemented with G418 (1mg/ml) and the growth medium was changed every three or four days. Following G418 selection for two weeks, G418 resistant clones were trypsinized, and 1/3 of the cells were transferred to 96-well PCR plates for PCR screening.

*PCR screenings*

G418-resistant cells in 96-well PCR plates were centrifuged and re-suspended in 25 μl lysis buffer (50 mM KCl, 1.5 mM MgCl2, 10 mM Tris-Cl, pH 8.5, 0.5% Nonidet P40, 0.5% Tween, 400 μg/ml Proteinase K). The cells were lysed at 65°C for 30 min followed by 95°C for 10 min. One μl lysate was used for PCR screening. Primers for screening of the BRCA1 targeting events are listed in Table S1. PCR conditions were as follows: (1) Neo-PCR: 1 cycle of 94°C for 2 min; 35 cycles of 94°C for 20 s, 67°C for 30 s, and 68°C for 40 s; followed by 68°C for 7 min. (2) BRCA1-KO-PCR: 1 cycle of 94°C for 2 min; 35 cycles of 94°C for 20 s, 61°C for 30 s, and 68°C for 3 min; followed by 68°C for 7 min. (3) 5’-AAV-RI-PCR and 3’-AAV-RI-PCR: 1 cycle of 94°C for 5 min; 35 cycles of 94°C for 30 s 68 for 30 s, and 68°C for 2.5 min; followed by 68°C for 7 min. All PCRs in this study were carried out using Platinum Pfx DNA Polymerase (Cat no. 11708-039, Invitrogen) in Applied Biosystems 96-Well GeneAmp(r) PCR System 9700.
