# Supplementary material for: An improved PCR strategy for fast screening of specific and random integrations in rAAV-mediated gene targeted cell clones
Source: BMC Res Notes. 2011 Jul 21;4:246. doi: 10.1186/1756-0500-4-246 (PMC3154164; doi:10.1186/1756-0500-4-246)
Supplement: Additional file 2 — Primers. list of primers used in this study. [file 1756-0500-4-246-S2.DOC]

**Additional file 2**

**Table S1** List of primers used in this study

| Name | Sequences (5’🡪3’) |
| --- | --- |
| F1 | TAGCAAAGAGCCAACAGAGCAGATG |
| F2 | ACTCCATCACTAGGGGTTCCTGC |
| F3 | GAAGCCCGGCATTCTGCACGC |
| F4 | CTCGCTGATCAGCCTCGACTG |
| R1 | AGGTAGCCGGATCAAGCGTATGCAG |
| R2 | ACTCCATCACTAGGGGTTCCTGC |
| P1 | atgagggtcactggccattaagtc |
| P2 | TTATGGCACTCAGGACAGTATCTC |

Note: F2 is located in the ITR region, and it is the same primer as R2.
